# Forty sites of TRP channel regulation

**DOI:** 10.1016/j.cbpa.2024.102550

**Published:** 2024-11-30

**Authors:** Irina A. Talyzina, Kirill D. Nadezhdin, Alexander I. Sobolevsky

**Affiliations:** Department of Biochemistry and Molecular Biophysics, Columbia University, New York, NY, USA

**Keywords:** TRP channels, Cryo-EM, X-ray crystallography, Gating, Activation, Inhibition, Block, Ligand, Agonist, Antagonist

## Abstract

Transient receptor potential (TRP) channels are polymodal molecular sensors that integrate chemical, thermal, mechanical and electrical stimuli and convert them into ionic currents that regulate senses of taste, smell, vision, hearing, touch and contribute to perception of temperature and pain. TRP channels are implicated in the pathogenesis of numerous human diseases, including cancers, and represent one of the most ardently pursued drug targets. Recent advances in structural biology, particularly associated with the cryo-EM “resolution revolution”, yielded numerous TRP channel structures in complex with ligands that might have therapeutic potential. In this review, we describe the recent progress in TRP channel structural biology, focusing on the description of identified binding sites for small molecules, their relationship to membrane lipids, and interaction of TRP channels with other proteins. The characterized binding sites and interfaces create a diversity of druggable targets and provide a roadmap to aid in the design of new molecules for tuning TRP channel function in disease conditions.

## Introduction

The superfamily of cation-selective TRP channels includes seven subfamilies: TRPV (vanilloid, TRPV1–6), TRPC (canonical, TRPC1–7), TRPM (melastatin, TRPM1–8), TRPN (NOMPC-like), TRPA (ankyrin, TRPA1), TRPP (polycystin, TRPP1–3) and TRPML (mucolipin, TRPML1–3). We will discuss structural pharmacology of all subfamilies, except TRPN, for which the available structural information is limited.

## Structural architecture of TRP channel subfamilies

**Figure 1:**
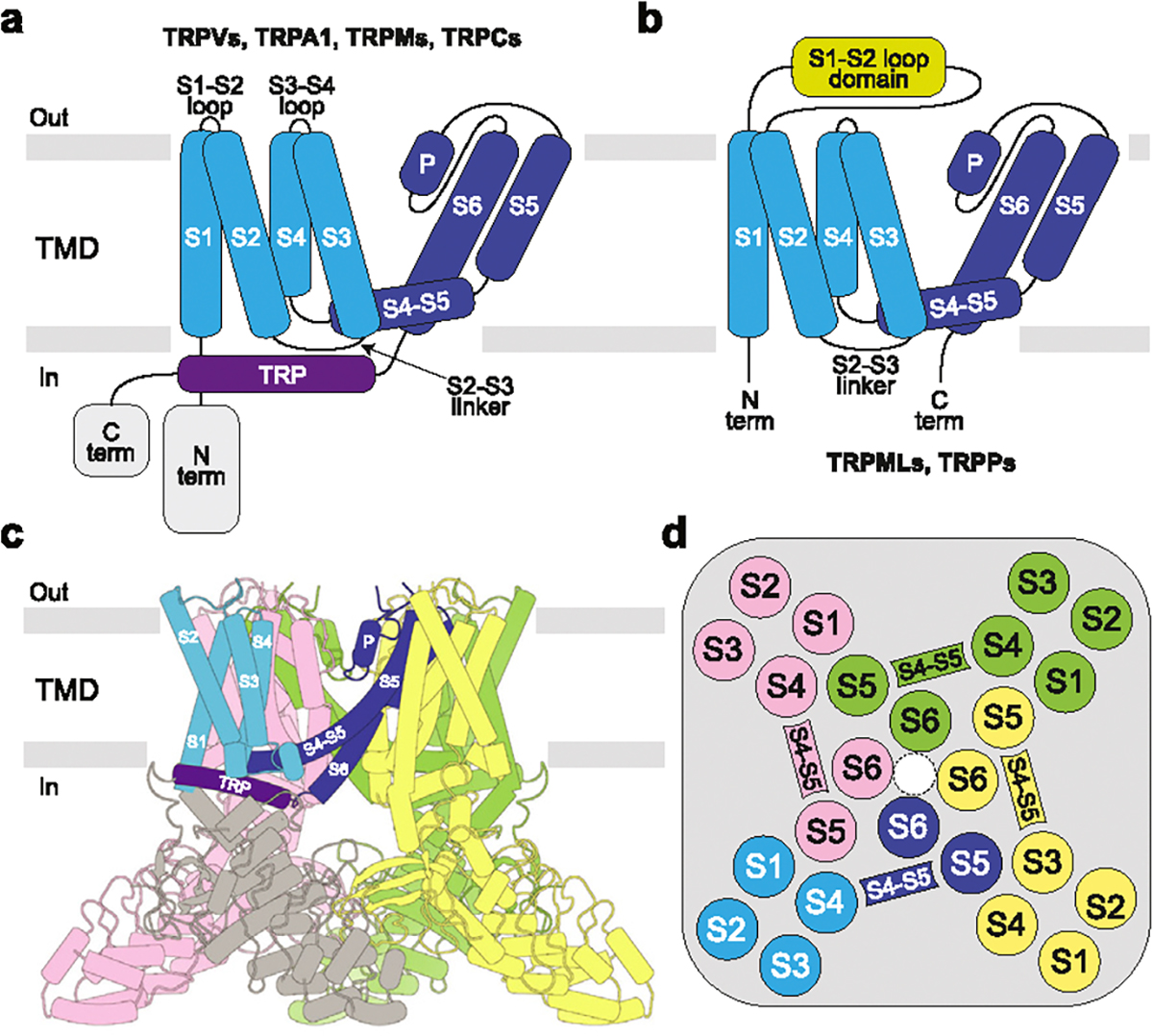
TRP channel structural architecture. **(a-b)**, Membrane topology for TRPV, TRPA, TRPM, and TRPC **(a)** as well as TRPML and TRPP **(b)** subfamilies of TRP channels, with TRP helix, intracellular, TMD segments, and S1–S2 domains labeled. **(c)**, Structure of TRPV1 (PDB ID: 8GF9) with four subunits colored differently and TMD segments colored as in **(a,b)**. **(d)**, Arrangement of TMDs viewed extracellularly and colored as in **c.** Note swapping of the S1–S4 and pore (S5–P-loop–S6) domains.

TRP channels are tetramers, with individual subunits composed of the intracellular N- and C-terminal domains and the transmembrane domain (TMD) in the middle (Figure 1). TMD is the most conserved region across the TRP channel superfamily, which includes six transmembrane (TM) helices (S1–S6) and a reentrant pore loop (P-loop) between S5 and S6, followed by the family signature domain–TRP helix. The first four TM helices comprise the S1–S4 or voltage sensing-like domain (VSLD). Four S1–S4 domains surround the ion channel pore in the center of the TMD, contributed by the pore domains (S5, P-loop and S6) of all four subunits. The N- and C-terminal domains are subfamily-specific and entirely absent in TRPP and TRPML channels, which also lack the TRP-helix but instead have extended S1–S2 loops that form polycystin or mucolipin domains, respectively.

## Ligand binding sites

**Figure 2:**
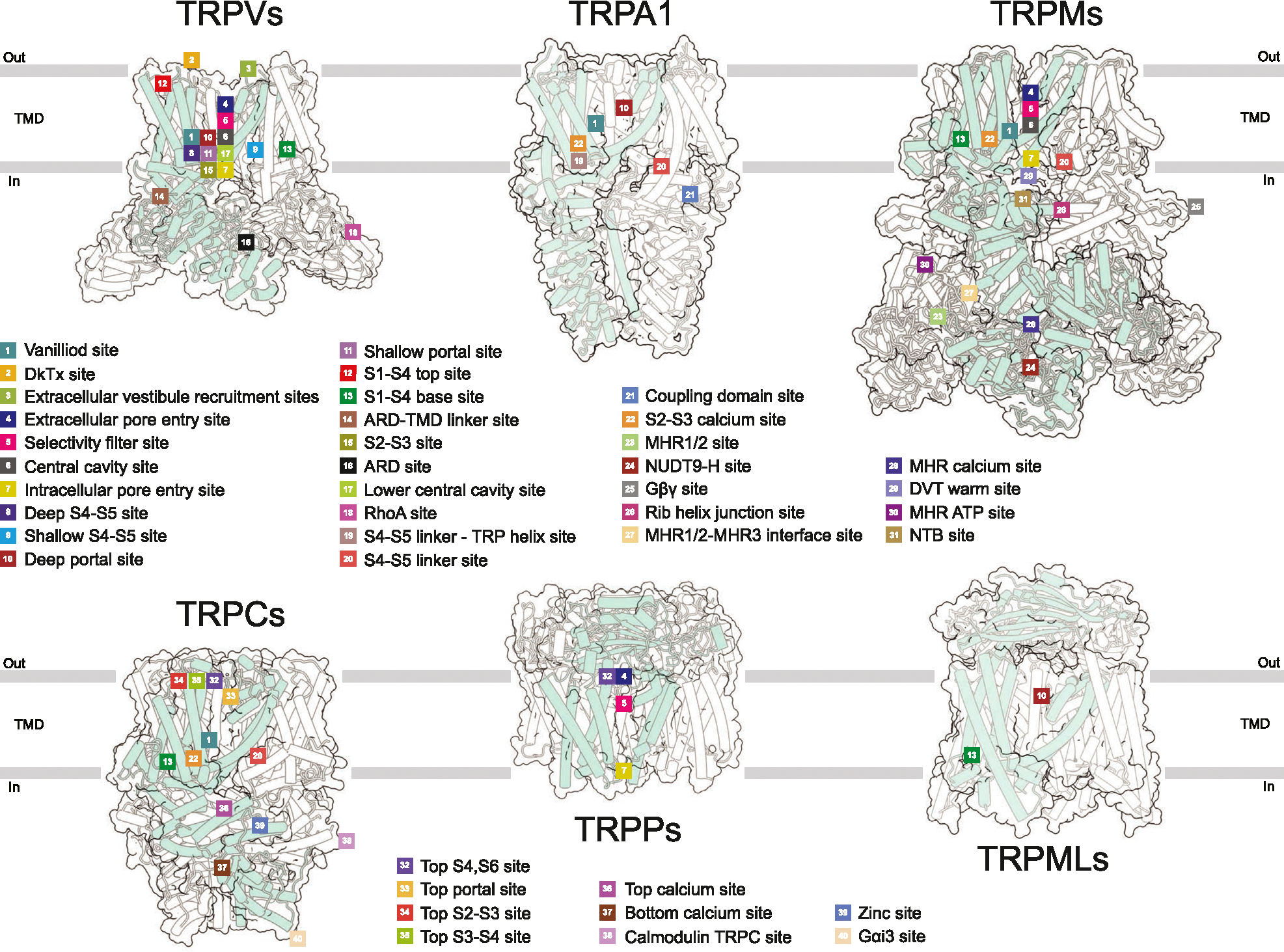
Ligand binding sites in different members of TRP channel superfamily. TRP channel subfamilies are represented by structures of TRPV1 (PDB ID: 8GF8, vanilloid subfamily), TRPA1 (PDB ID: 6PQQ, ankyrin subfamily), TRPM2 (PDB ID: 6PUO, melastatin subfamily), TRPC5 (PDB ID: 7WDB, canonical subfamily), TRPP2 (PDB ID: 8HK7, polycystin subfamily), and TRML1 (PDB ID: 7SQ8, mucolipin subfamily). Ligand-binding sites are mapped on the corresponding structures, labeled numerically and annotated.

Structural studies characterized the interaction of ligands with specific regions of the TRP channel proteins which can be classified as forty distinct ligand binding sites, based on their location with respect to the conserved structural elements (Figure 2, Supplementary Table 1). These sites are located either within the individual domains (N- or C-terminal or TMD) or at the domain interfaces. The most common binding sites, which have been reported for several TRP channel subfamilies, are vanilloid (#1), S1–S4 base (#13), portal (#10–11), S4–S5 linker (#19–20) and S2–S3 calcium (#22) sites. All five have dual functionality and can bind both activators and inhibitors.

The vanilloid site (*#*1) is located at the interface between the S1–S4 and pore domains and contributed by the S4–S5 linker, S3, S4 and TRP helices of one subunit and S5 and S6 helices of the adjacent subunit [1]. Both agonists (capsaicin and resiniferatoxin) and competitive antagonists (capsazepine, SB-366791, and SAF312) can occupy this site in TRPV1. The agonists engage Arg557 into a salt bridge with Glu570, pulling S5 towards S1–S4 domain and leading to channel opening. In contrast, the antagonists disfavor the Arg557-Glu570 interaction and stabilize the closed state [2,3]. The vanilloid site also binds phosphoinositol lipids, lysophosphatidic acid, and cholesterol in TRPV1; agonist 2-APB and cholesterol in TRPV2; agonist THCV and inhibitor Trpvicin in TRPV3; cholesteryl hemisuccinate (CHS) and channel blockers PCHPDs in TRPV6; inhibitor BTDM in TRPC6; antagonist NDNA in TRPM5; activator AITC in TRPM8; inhibitors CCT128930, NS8593, VER155008, and activator naltriben in TRPM7; and non-covalent agonist GNE551 in TRPA1 (Supplementary Table 1).

The S1–S4 base (#13) site represents a cavity formed by the intracellular ends of all four helices of the S1–S4 bundle and the TRP helix [1]. In the absence of added ligands (apo condition), this site in TRPV subfamily representatives is typically occupied by a lipid. The S1–S4 base site also binds activator 2-APB and inhibitor osthole in TRPV3; agonists 4α-PDD and Agonist1, and antagonists A1, A2, GSK1016790A, and HC-067047 in TRPV4; inhibitor ZINC17988990 in TRPV5; inhibitor 2-APB in TRPV6; agonists Cryosim-3, icilin, and WS-12 in TRPM8; inhibitors GFB-8438, GFB-8749, and GFB-9289 in TRPC4; activator riluzole in TRPC5; antagonist AM-1473 and SAR7334 in TRPC6; and phospholipids PI(3,5)P_2_ and PI(4,5)P_2_ in TRPML1 (Supplementary Table 1).

The portal site (#10–11) is the closest to the pore site located at the intersubunit interface, contributed by S5 of one subunit as well as P-loop and S6 of another subunit [1]. The portal site accommodates cholesterol in TRPV1; activator CBD in TRPV2; inhibitor dyclonine in TRPV3; inhibitor THCV in TRPV6; inhibitor GDC-0334 in TRPA1; inhibitors HC-070 and Pico145 (HC-608) in TRPC5; as well as agonists ML-SA1 and temsirolimus, and inhibitor ML-SI3 in TRPML1 (Supplementary Table 1). The S4–S5 linker site (#19–20) is contributed by S1, S4–S5 linker and TRP helix as well as the linker domain in TRPC and TRPM channels or interfacial helix in TRPA1. The S4–S5 site accommodates the antagonist 3–60 in TRPA1; CHS in TRPC3–6 and TRPM8; and PI(4,5)P_2_ in TRPM3 and TRPM8 (Supplementary Table 1).

The S2–S3 calcium site (#22) represents a common site for Ca^2+^ ion in TRPA1, TRPC3-TRPC6, TRPM2, TRPM4–5, and TRPM8 (Supplementary Table 1). It is comprised of four residues (D, E, N, or Q in different combinations) that belong to intracellular regions of S2 and S3. Ca^2+^ ion bound to the S2–S3 site can either have the activating (TRPC3,6 and TRPM4,5) or regulatory (TRPA1, TRPM2,8) roles, either enhancing ligand binding or desensitization [4,5]. Ca^2+^ is displaced from the S2–S3 site in TRPC6 by the inhibitors AM-1473 [6] and SAR7334 [5]. On the other hand, Ca^2+^ helps to coordinate the activator riluzole in TRPC5 [7] and cooling agonist icillin in TRPM8 [8].

While different ligands — both activators and inhibitors — can bind to a single site, the same ligand can bind to different sites in one ion channel simultaneously. For example, 2-APB binds to three distinct sites in TRPV3 (#12, #13, #14), but it also binds to TRPV2 (#1, #9) and TRPV6 (#13) [1,9,10]. Naltriben binds to two different sites in TRPM7, resulting in either channel activation (NTB site, #31) or inhibition (vanilloid site, #1) [11]. Strikingly, recent structural studies of TRPM4 showed that decavanadate (DVT) binding is temperature-dependent, occurring either at the MHR1/2-MHR3 (#27) or warm DVT (#29) sites [12] (Supplementary Table 1). In addition, different sites can be occupied by different ligands, which activate or inhibit TRP channels cooperatively or synergistically [13–17]. For instance, the activation of TRPV2 by 2-APB and CBD applied simultaneously is much stronger than the cumulative activation produced by these agonists applied individually [16].

## Lipid binding sites

**Figure 3:**
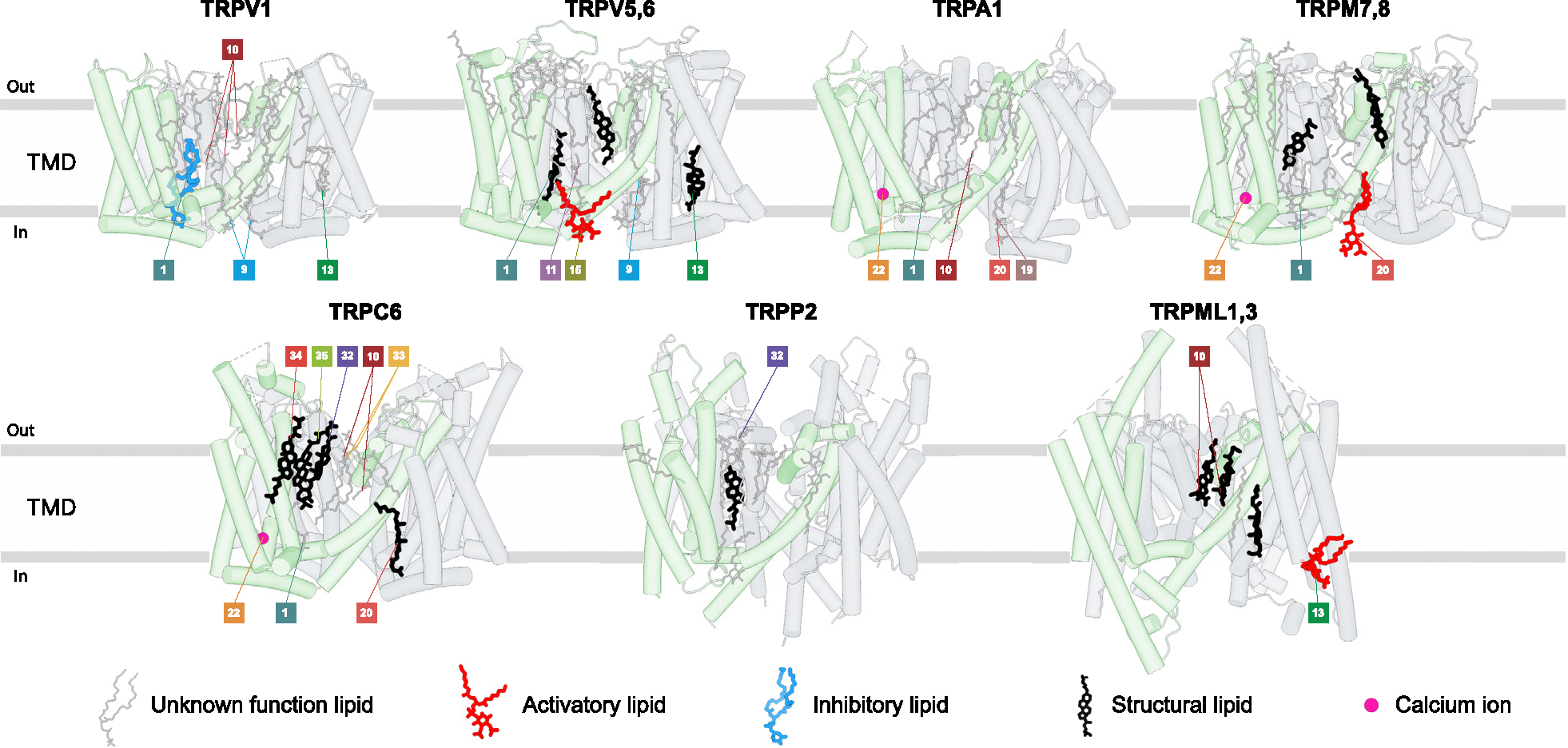
Structurally resolved annular lipids. Cartoons show transmembrane regions for TRP channel representatives, including TRPV1 (PDB ID: 8GF9), TRPV5,6 (PDB ID: 7S88, 6DMU) TRPA1 (PDB ID: 6PQQ, 6V9W), TRPM7,8 (PDB ID: 8SI2, 8E4M), TRPC6 (PDB ID: 7DXG, 6UZA, 7DXF), TRPP2 (PDB ID: 6T9N), and TRML1,3 (PDB ID: 5W3S, 7WDB). The annular lipids are shown as sticks and color coded as activating (red), inhibiting (blue), structural (black) and other (grey). Calcium ions that bind in the TMD are shown as pink spheres.

In the apo state, many ligand-binding sites are occupied by annular lipids, which in the high-resolution cryo-EM structures of TRP channels are represented by non-protein densities around the TMD. Some lipids are required for protein stability (structural lipids) [18,19], while others modulate channel function [20], playing activatory or inhibitory roles (Figure 3). The two classes of lipid molecules that are commonly recognized in cryo-EM structures due to their distinct features are cholesterol and phospholipids.

Densities for cholesterol or its more soluble acidic ester CHS, often used in protein purifications, have a clearly recognizable flat shape of sterol with holes and bumps corresponding to cyclohexane rings and methyl groups, respectively. Among the vanilloid-subfamily TRP channels, temperature-insensitive calcium-selective TRPV5–6 appear to require cholesterol or CHS for their structural integrity, as every structure of these channels show structural lipids at the ligand-binding sites #1 (vanilloid), #13 (S1–S4 base), and #33 (top portal) (Figure 3). In temperature-sensitive TRPV channels, on the other hand, only one site (#1, vanilloid) was reported to bind endogenous cholesterol in TRPV2 [10] and two sites (deep portal [19] and adjacent to vanilloid [13] sites) in TRPV1. Cholesterol was also reported to sensitize TRPV3 [21] and regulate function, expression, and the cellular response to tension of TRPV4 [22]. However, the corresponding sites of cholesterol binding to TRPV3 and TRPV4 await structural determination. While CHS is frequently used as a substitute for endogenous cholesterol, it is important to keep in mind that the hydrophilic hemisuccinate group in CHS versus the hydrophobic hydrocarbon group in cholesterol may cause differences in effects of these lipids on certain TRP channels.

Densities for phospholipids have a distinct head-and-two-tails appearance. The head density of the majority of phospholipids (excluding inositol phospholipids) has an elongated cylindrical shape, making it difficult to distinguish between different types of phospholipids. Accordingly, while many putative phospholipids consistently appear in different TRP channel structures, assigning their structural or functional roles is often difficult due to unclear chemical identity of the lipid and uncertain binding site specificity. In rare cases, the functional role of phospholipids becomes apparent when their site occupancy changes during gating. An example is a phospholipid in TRPV3, which occupies the vanilloid site (#1) in the closed state but must leave this site for the channel to open in response to heat [23,24] or agonist binding [9]. For inositol phospholipids, the head density resembles a donut, with additional bumps representing the number of phosphate groups attached to the inositol ring. Phosphatidylinositol lipids are known to regulate many TRP channels, serving as essential cofactors for their activity [20]. Thus, TRPV1 has a residential phosphatidylinositol lipid that is crucial for its stability and defines the temperature activation threshold and affinity to ligands [19]. PI(4,5)P_2_ was shown to activate all TRPM subfamily members except TRPM1 [20], with structures available for TRPM8 and TRPM3 [8,17,25,26]. On the other hand, the intracellular TRPML1 channel is inhibited by PI(4,5)P_2_ and activated by PI(3,5)P_2_, both binding to the same site [14,27]. PI(4,5)P_2_ was reported necessary for TRPV1–4, TRPA1, and TRPC4–5 activity but also as their inhibitor [20,28]. Structures of these channels bound to PI(4,5) P_2_ should untangle their complex interaction.

While several ligand-binding sites host lipids in the apo condition (Figure 3), many lipid binding locations have not been identified as sites for ligands. We anticipate future discoveries of ligands targeting the corresponding protein-lipid interfaces and acting by outcompeting endogenous lipids.

## TRP channel protein binding partners

**Figure 4:**
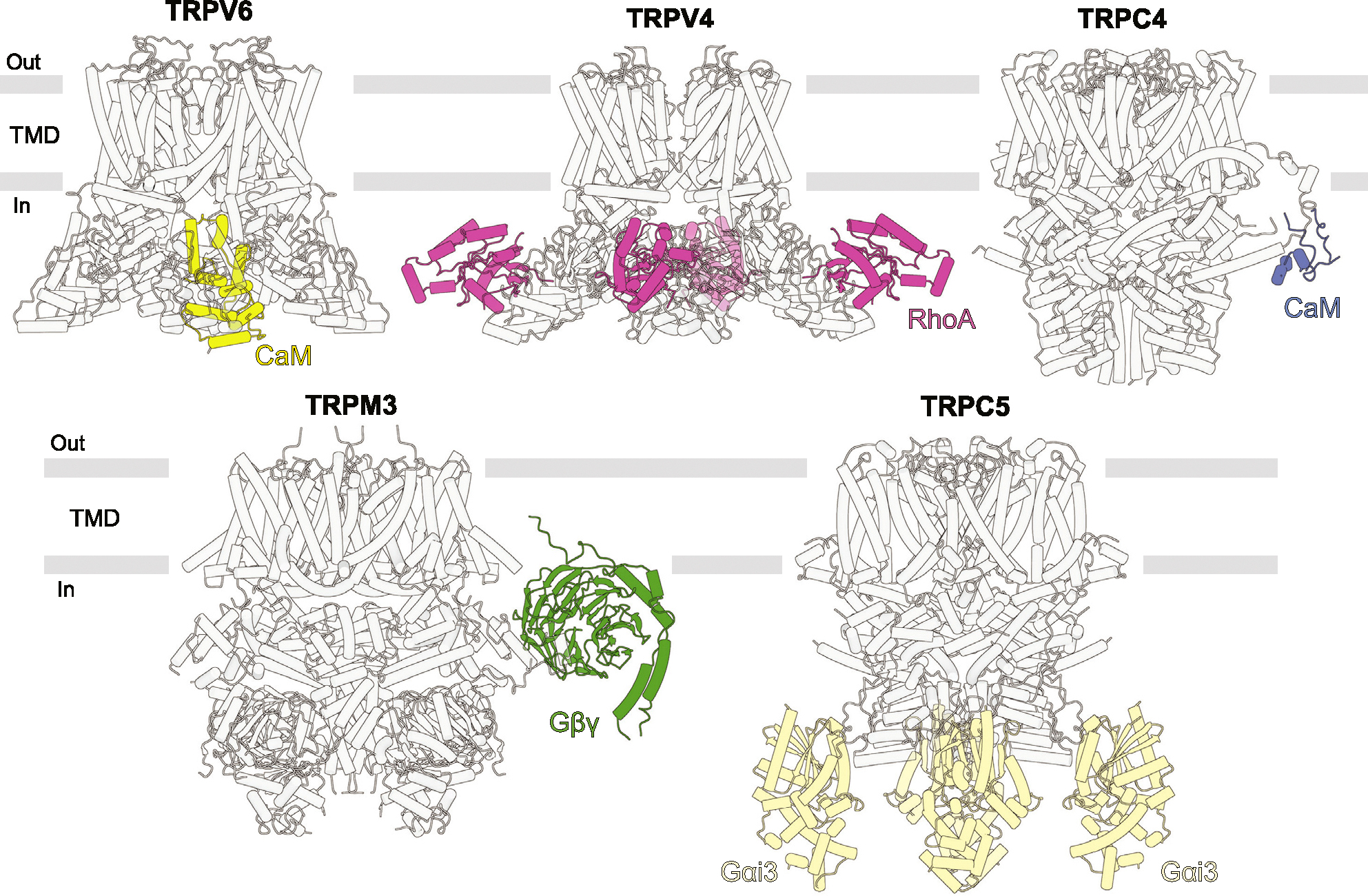
Interactions of TRP channels with other proteins. Shown are structures of TRPV6 in complex with CaM (PDB ID: 6E2F), TRPV4 in complex with RhoA (PDB IDs: 8T1B and 8T1C), TRPC4 in complex with CaM (PDB ID: 7B1G), TRPM3 in complex with Gβγ (PDB ID: 8DDX), and TRPC5 in complex with Gαi_3_ (PDB ID: 7X6I). TRP channels are shown in grey, while their binding partners in colors of the corresponding binding sites (Figure 2).

In addition to small molecules, the function of ion channels in general, and TRP channels in particular, is often regulated by other proteins, which represent either transmembrane auxiliary subunits or soluble binding partners. Cryo-EM structures have been recently solved for complexes of TRPV5/6 with CaM [29–32], TRPV4 with RhoA [33–35], TRPC4 with CaM [36], TRPM3 with Gβγ [25], and TRPC5 with Gα_i3_ [37] (Figure 4).

The TRPV5/6-CaM structures reveal the mechanism of CaM-mediated inactivation as a pore block by K115 of CaM, which forms a unique cation-π interaction with a cubic cage of four tryptophan indole rings at the ion channel intracellular entrance. The structures of TRPV4-RhoA were captured in different conformations of TRPV4 but showed no difference in RhoA binding, providing limited information about the mechanism of RhoA-mediated TRPV4 inhibition. RhoA, which is known to undergo a posttranslational modification by prenylation, can be anchored to the plasma membrane through its prenylated C-terminus. This interaction may enable TRPV4 to sense alterations in cellular shape and morphology resulting from osmotic shock or mechanical forces [33]. Like TRPV5–6, TRPC4 undergoes inactivation by CaM [36]. In the TRPC4-CaM structure, CaM binds to the rib helix of TRPC4. The authors proposed that this binding stabilizes a previously disordered region, which is directly connected to the TRP helix, restricts its mobility and allosterically locks the channel in the closed state.

The structure of TRPM3-Gβγ was solved in the closed state. Understanding the mechanism of TRPM3 inhibition by Gβγ will require solving the open-state structure of TRPM3. Similarly, Gα_i3_ is known to potentiate TRPC5 [37] but the TRPC5-Gα_i3_ structure is solved in the closed state. Likewise, solving the structure of TRPC5-Gα_i3_ in the open state will be necessary to understand the mechanism of TRPC5 potentiation by Gα_i3_. Similar to the TRPV4-RhoA interaction, the action of Gα_i3_ and Gβγ might be dependent on posttranslational modifications of these G proteins by lipidation [25,37]. Lipidation may indirectly affect TRP channels by increasing the local concentration of G proteins near membrane and causing clustering of TRP channels.

In particular cellular conditions, interaction of TRP channels with protein binding partners may depend on additional factors. One such factor is the functional state of the binding partner. For example, GTPase RhoA in its inactive, GDP-bound form can inhibit TRPV4 [38], while activated, GTP-bound Gα_i3_ can potentiate TRPC5 [37]. Another important factor is the concentration of second messengers. Ca^2+^ binding to both CaM and TRPC5 is required for inactivation of TRP channels by CaM and for the TRPC5-Gα_i3_ complex formation [29–32,36,37,39]. PIP_2_ has been reported as a stabilizing factor for the TRPC5-Gα_i3_ and TRPM3-Gβγ interactions [25,37]. Many interactions of TRP channels with other proteins, like TRPV3 with Ano1 [40] or TMEM79 [41], and their regulation by different cofactors remain elusive and will require additional structural studies to understand the corresponding molecular mechanisms.

## Conformational changes caused by ligand binding

**Figure 5:**
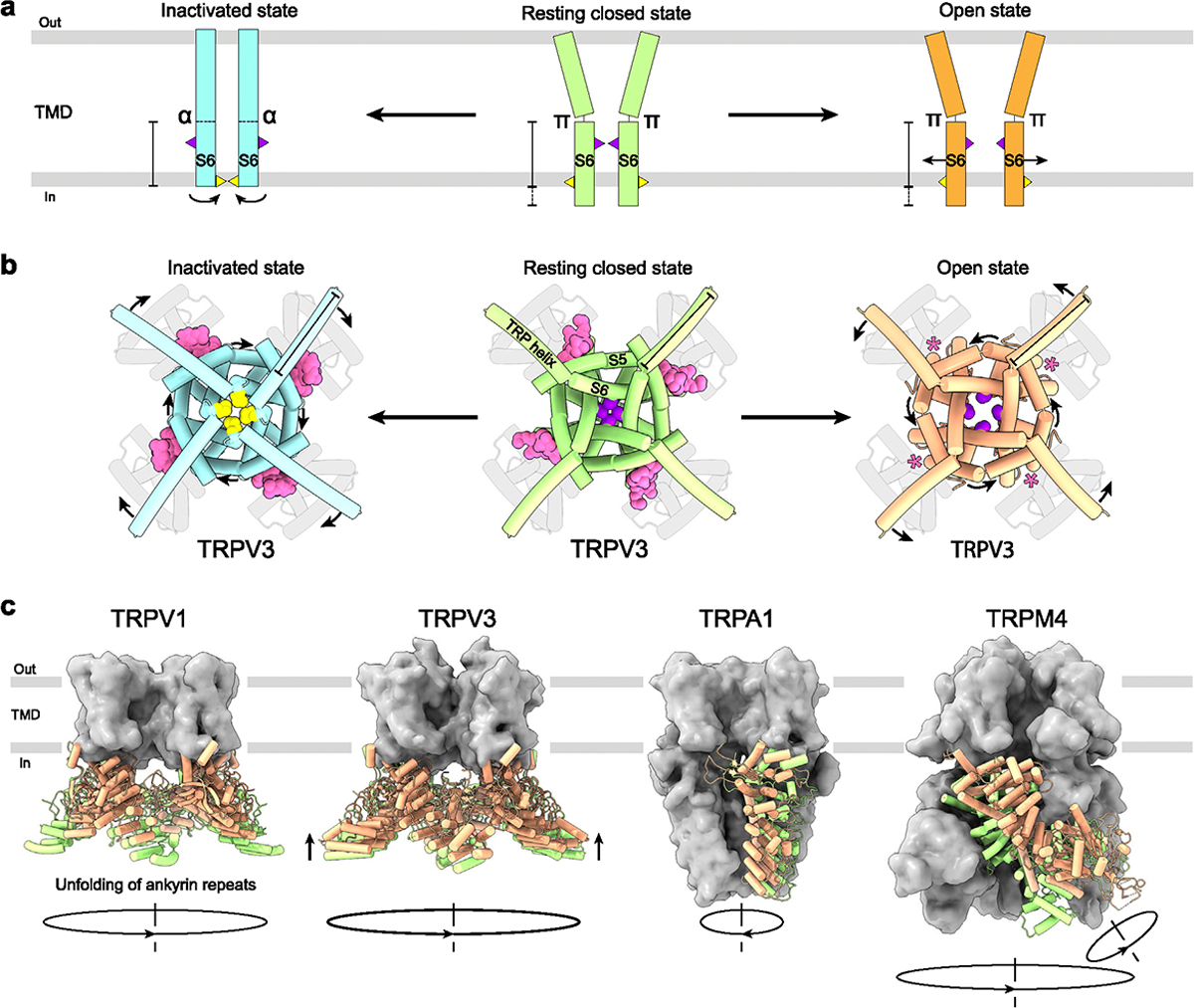
TRP channel conformational changes in response to ligand binding. **(a).** Schematic transitions in S6 that occur during TRP channel opening and inactivation. The closed state structure is shown in green, open in orange, and inactivated in light blue. **(b).** Structures of human TRPV3 in the closed resting (PDB ID: 8V6K), 2-APB-bound open (PDB ID: 8V6N) and inactivated (PDB ID: 8V6O) states, with the length of the TRP helices in the closed/open states and their rotation during opening and inactivation indicated. The vanilloid site lipid is pink, I674 violet and M677 yellow. **(c).** Superposed are structures of TRPV1 (PDB IDs: 7RQW, 7RQU), TRPV3 (PDB IDs: 8V6K, 8V6L), TRPA1 (PDB IDs: 6V9W, 6V9X), and TRPM4 (9B93, 9B8Y) in the closed (green) and open (orange) states. Movements of intracellular domains during opening are indicated by arrows.

Numerous TRP channel structures solved in complex with ligands have revealed conformational changes and molecular mechanisms associated with channel regulation and gating. Nevertheless, a substantial number of TRP channel structures in complex with activators or inhibitors have been solved in apo-like states, where protein conformational changes occur only locally, in close proximity to the bound ligands. Typically, such structures are less informative and require functional experiments combined with mutagenesis or MD simulations to gain insights into the mechanism of ligand action. When ligands do induce global conformational changes, they typically resolve one or two new states of the channel conformational ensemble distinct from the closed resting (apo) state: the ligand-bound open state with the conducting pore and/or ligand-bound inhibited or inactivated/desensitized state with the closed pore (Figure 5a and b).

The most pronounced conformational changes associated with TRP channel gating typically happen in the S4eS5 linker, S5, the N-terminal part of the TRP helix, and most importantly S6, which forms the channel gate. When channel pore dilates during opening, S6 moves away from the pore center (Figure 5a). Meanwhile, the N-terminal part of the TRP helix tilts to compensate for the movement of S6 (Figure 5b). This tilt of TRP helix and movement of S6 helix during the closed-to-open transition in vanilloid (V), melatonin (M), and mucolipin (ML) subfamilies results in counterclockwise rotation of the pore-forming helices S5 and S6 around the pore axis when viewed intracellularly (Figure 5b). Importantly, since no open-state structures have been solved for TRPC and TRPP subfamilies, whether these channels follow the same pattern of conformational changes during opening remains unclear.

Inactivation, on the other hand, is often linked to secondary structure changes in S6 itself. Thus, in TRPV3, inactivation is accompanied by a ~100° rotation of the C-terminal portion of S6 converting it from a π-bulge-containing to entirely α-helical (Figure 5a). The rotation in S6 exposes a completely different set of residues to the channel pore, with methionines M677 and isoleucines I674 forming the narrowest part of the human TRPV3 pore in the inactivated and closed/open states, respectively [9]. In addition, inactivation is accompanied by a two helical turns shortening of S6 and two helical turns elongation of the TRP helix (Figure 5a and b). Similar transitions were observed in TRPV1, TRPV4, and TRPM2 in response to binding of agonists or inhibitors [19,34,42,43]. Despite this similarity, conformational changes that happen upon inactivation in the majority of other TRP channels do not follow the same pattern [4,5,15,29,30,32,33,35,44–49]. For example, the closed state of TRPV6 is characterized by α-helical S6, while the open and inactivated states have S6 with the π-bulge in the middle [50]. In addition, structures reported in intermediate [19,24,51,52] and non-canonical pentameric [53] conformations indicate that the 3-state model of TRP channel gating (Figure 5a and b) is an obvious oversimplification, signifying the need of developing more complete and sophisticated gating models.

The global structural rearrangements during activation are unique for each TRP channel subfamily and characterized by distinct architectural features (Figure 2). In TRPV1 channel, pore opening is accompanied by unfolding of the N-terminal ankyrin repeat domains (ARDs). During TRPV3 activation, the intracellular skirt domain moves towards the TMD and rotates 8° clockwise around the fourfold rotational symmetry axis when viewed intracellularly, while four C-termini unwrap and form α-helices [9]. Opening of the TRPM4 channel involves a 52°-rotation of the N-terminal melastatin homology region (MHR) domains [12] (Figure 5c). In contrast to TRPV, TRPM, and TRPML channels, activation of TRPA1 is accompanied by movement of S5–S6, TRP helices, and N-terminal domains in the opposite direction, clockwise when viewed intracellularly [54] (Figure 5c). The molecular mechanisms guiding these global conformational rearrangements, and a possible role of currently unresolved structural domains await future structural studies.

## Conclusion

In this review, we provided an update on the current status of TRP channel structural pharmacology and classified 40 discovered binding sites that regulate TRP channel function. We highlight the importance of lipids and ions in modulating channel function and describe commonly observed conformational changes induced by ligand binding. Given modern advances in structural biology, we anticipate discovery of many more new sites, which will aid in drug design for treatment of TRP channel-related diseases.

## Supplementary Material

MMC1

## Data Availability

No data was used for the research described in the article.
