# Supplementary material for: Forty sites of TRP channel regulation
Source: Curr Opin Chem Biol. Author manuscript; Available in PMC 2025 Feb 3. (PMC11788071; doi:10.1016/j.cbpa.2024.102550)
Supplement: MMC1 [file NIHMS2035709-supplement-MMC1.docx]

**Supplementary Table 1. Binding sites in TRP channels**

| **Site #** | **Site name** | **Ligands** | **Channel** | **Function** | **Affinity (μM)** | **Ref. affinity** | **PDB IDs** | **Ref. PDB IDs** |
| --- | --- | --- | --- | --- | --- | --- | --- | --- |
| **TRPV sites** | | | | | | | | |
| **1** | Vanilliod site | Capsaicin | Rat TRPV1 | Agonist | EC_50_ 0.2-1.9 | [55-58] | 3J5R 7LPA 7LPB 7LPD 7LPE | [52,59] |
| **1** | Vanilliod site | Capsaicin | Squirrel TRPV1 | Agonist | EC_50_ 0.53 ± 0.03 | [56] | 7LR0 | [42] |
| **1** | Vanilliod site | RTX | Rat TRPV1 | Agonist | EC_50_ 0.007-0.1 | [55,57,60,61] | 7MZE  5IRX  7L2L  7L2M  7L2N  7L2O  7L2V  7L2W  7L2X  7MZ5  7MZ7  7MZ9  7MZC  7MZD  7RQU  7RQV  7RQW  7RQX  7RQY  7RQZ | [2,51,62] |
| **1** | Vanilliod site | RTX | Squirrel TRPV1 | Agonist | Working concentration: 50 | [42] | 7LQZ | [42] |
| **1** | Vanilliod site | RTX | Rabbit TRPV2 | Agonist | Working concentration: 0.25 | [63] | 6BWJ | [63] |
| **1** | Vanilliod site | Capsazepine | Rat TRPV1 | Competitive antagonist | IC_50_ 0.3 - 2.4 | [57,60,64] | 5IS0 | [2] |
| **1** | Vanilliod site | PI | Human TRPV1 | Negative regulator | Endogenous | - | 8GF8 8GF9 | [3] |
| **1** | Vanilliod site | PI | Rat TRPV1 | Negative regulator | Endogenous | [65] | 8T0Y  8T10  8T3L  8T3M  5IRZ  7L2H  7L2I  7L2J  7L2P  7L2R  7L2S  7L2T  7L2U  7LP9  7LPC  7MZ6  7MZA  7MZB  8T0E  8U3A  8U3C | [2,19,52,62] |
| **1** | Vanilliod site | PI | Squirrel TRPV1 | Negative regulator | Endogenous | - | 7LQY | [42] |
| **1** | Vanilliod site | DiC8-PI(4,5)P_2_ | Rat TRPV1 | Partial potentiator | Working concentration: 50 | [19] | 8U2Z  8U30 | [19] |
| **1** | Vanilliod site | Di18:1 PI(4,5)P_2_ | Rat TRPV1 | Negative regulator | Working concentration: 10% | [65] | 8U43 | [19] |
| **1** | Vanilliod site | Lysophosphatidic acid (LPA) | Rat TRPV1 | Agonist | EC_50_ 0.754 | [66] | 8T0C  8T0Y  8T10  8T3L  8T3M | [19] |
| **1** | Vanilliod site | Cholesterol | Human TRPV1 | Coregulator | Endogenous | [13] | 8JQR  8X94 | [13] |
| **1** | Vanilliod site | Cholesterol | Mouse TRPV2 | Inhibitor | IC_50_ 74.6 ± 25.1 | [10] | 7XEM 7XER | [10] |
| **1** | Vanilliod site | CHS | Human TRPV6 | Structural lipid | Working cocnentration: 0.001-0.1% | [47,67] | 7K4A  7S88  7S89 | [47,67] |
| **1** | Vanilliod site | Econazole | Rabbit TRPV5 | Inhibitor | IC_50_ 1.3-13 | [68-70] | 6B5V | [68] |
| **1** | Vanilliod site | PCHPD (Br-cis-22a) | Rat TRPV6 | Inhibitor | IC_50_ 0.96 ± 0.03 | [67] | 7D2K | [67] |
| **1** | Vanilliod site | PCHPDs (cis-22a, Br-cis-22a, 3OG, 30, 31) | Human TRPV6 | Inhibitor | IC_50_ 0.08 - 1.7 | [67,71-73] | 7K4B 7K4C 7K4D 7K4E 7K4F | [67] |
| **1** | Vanilliod site | 2-APB | Mouse TRPV2 | Agonist | EC_50_ 1,424 ± 142.7 | [10] | 7XEV  7YEP | [10] |
| **1** | Vanilliod site | BTDM | Human TRPC6 | Inhibitor | IC_50_ 0.01 | [74] | 7DXF | [5] |
| **1** | Vanilliod site | AITC | Mouse TRPM8 | Agonist | Kd 2 900 ± 600 | [75] | 8E4L | [17] |
| **1** | Vanilliod site | CCT128930 | Mouse TRPM7 | Inhibitor | IC_50_ 1.42 ± 0.01 | [76] | 8W2L | [76] |
| **1** | Vanilliod site | GNE551 | Human TRPA1 | Non-covalent agonist | EC_50_ 0.207-0.312 | [77] | 6X2J | [77] |
| **1** | Vanilliod site | Naltriben | Mouse TRPM7 | Agonist | EC_50_ 65.8 ± 12.36 | [11] | 8SI6 | [11] |
| **1** | Vanilliod site | NDNA | Zebrafish TRPM5 | Antagonist | IC_50_ 0.0024 | [78] | 7MBV | [78] |
| **1** | Vanilliod site | NS8593 | Mouse TRPM7 | Inhibitor | IC_50_ 0.91±0.08 | [11] | 8SIA | [11] |
| **1** | Vanilliod site | SAF312 | Human TRPV1 | Non-competitive antagonist | IC_50_ 0.0073 - 0.69 | [13,79] | 8JQR  8X94 | [13] |
| **1** | Vanilliod site | SB-366791 | Human TRPV1 | Inhibitor | IC_50_ 0.021 ± 0.006 | [3] | 8GFA | [3] |
| **1** | Vanilliod site | THCV | Human TRPV3 | Agonist | EC_50_ 6.1  ±  0.5 | [9] | 8V6L  8V6M | [9] |
| **1** | Vanilliod site | Trpvicin | Human TRPV3 | Antagonist | IC_50_ 0.41 | [80] | 7XJ0  7XJ1  7XJ2 | [80] |
| **1** | Vanilliod site | VER155008 | Mouse TRPM7 | Inhibitor | IC_50_ 0.23 ± 0.03 | [11] | 8SI7  8SI8 | [11] |
| **2** | DkTx site | DkTx | Rat TRPV1 | Irreversible agonist | EC_50_ 0.14-0.24 | [81] | 3J5Q  5IRX  7L2M  7L2R  7L2S  7L2T  7L2U | [2,59,62] |
| **3** | Extracellular vestibule recruitment sites | Ca^2+^ | Rat TRPV6 | Permeant ion | EC_50_ 1470 ± 800 | [18] | 6D7P | [82] |
| **3** | Extracellular vestibule recruitment sites | Ba^2+^ | Rat TRPV6 | Permeant ion | EC_50_ 1910 ± 740 | [18] | 5IWR | [18] |
| **3** | Extracellular vestibule recruitment sites | Gd^3+^ | Rat TRPV6 | Channel blocker | IC_50_ 3.87 ± 0.83 | [18] | 5IWT  5WOA | [18,83] |
| **4** | Extracellular pore entry site | Ba^2+^ | Rat TRPV6 | Permeant ion | EC_50_ 1.91 ± 0.74 | [18] | 5IWR | [18] |
| **4** | Extracellular pore entry site | Ca^2+^ | Human PC2 | Agonist, channel blocker | Working concentration: 2 500 | [84] | 5MKF | [84] |
| **4** | Extracellular pore entry site | Ca^2+^ | Rat TRPV6 | Permeant ion | EC_50_ 1.47 ± 0.80 | [18] | 5IWK  5IWP  5WO9  6D7P | [18,83] |
| **4** | Extracellular pore entry site | Gd^3+^ | Rat TRPV6 | Channel blocker | IC_50_ 3.87 ± 0.83 | [18] | 5IWT  5WOA | [18,83] |
| **4** | Extracellular pore entry site | Mg^2+^ | Mouse TRPM7 | Permeant ion | Working concentration: 300 000 | [85] | 6BWD | [85] |
| **4** | Extracellular pore entry site | NMDG | Rat TRPV1 | Permeant ion | Working concentration: 28 000 | [62] | 7L2V  7L2W | [62] |
| **5** | Selectivity filter site | NMDG | Rat TRPV1 | Permeant ion | Working concentration: 28 000 | [62] | 7L2X | [62] |
| **5** | Selectivity filter site | Ba^2+^ | Frog TRPV4 | Permeant ion | Working concentration: 10 000 | [86] | 6C8G | [86] |
| **5** | Selectivity filter site | Ba^2+^ | Rat TRPV6 | Permeant ion | EC_50_ 1.91 ± 0.74 | [18] | 5IWR | [18] |
| **5** | Selectivity filter site | Ca^2+^ | Human PC2 | Agonist, channel blocker | Working concentration: 2 500 - 20 000 | [84,87] | 5MKE  5MKF  6T9N  6T9O | [84,87] |
| **5** | Selectivity filter site | Ca^2+^ | Human TRPV4 | Permeant ion | Working concentration: 2 000 | [88] | 7AA5 | [88] |
| **5** | Selectivity filter site | Ca^2+^ | Rabbit TRPV2 | Permeant ion | Working concentration: 2 000 | [63] | 6BWM | [63] |
| **5** | Selectivity filter site | Ca^2+^ | Rat TRPV6 | Permeant ion | EC_50_ 1.47 ± 0.80 | [18] | 5IWP  5WO6  5WO7  5WO8  5WO9 | [18,83] |
| **5** | Selectivity filter site | Cs+ | Frog TRPV4 | Permeant ion | Working concentration: 100 000 | [86] | 6C8F | [86] |
| **5** | Selectivity filter site | Gd^3+^ | Frog TRPV4 | Channel blocker | Working concentration: 2 000 | [86] | 6C8H | [86] |
| **5** | Selectivity filter site | Mg^2+^ | Mouse TRPM7 | Permeant ion | Working concentration: 300 000 | [85] | 6BWD | [85] |
| **5** | Selectivity filter site | Ruthenium Red | Human TRPV6 | Channel blocker | IC_50_ 9 ± 1 | [89] | 7S8B | [47] |
| **5** | Selectivity filter site | Ruthenium Red | Mouse TRPV4 | Channel blocker | IC_50_ 0.3 ± 0.1 | [45] | 8J1B | [45] |
| **5** | Selectivity filter site | Ruthenium Red | Rabbit TRPV5 | Channel blocker | IC_50_ 0.0137 ± 0.0044 | [90] | 8FFN  8FFQ | [90] |
| **5** | Selectivity filter site | Ruthenium Red | Rat TRPV2 | Channel blocker | IC_50_ 0.159 ± 0.047 | [90] | 8FFM | [90] |
| **6** | Central cavity site | Ba^2+^ | Rat TRPV6 | Permeant ion | EC_50_ 1.91 ± 0.74 | [18] | 5IWR | [18] |
| **6** | Central cavity site | Ca^2+^ | Rat TRPV6 | Permeant ion | EC_50_ 1.47 ± 0.80 | [18] | 5IWP  5WO9 | [18,83] |
| **6** | Central cavity site | Genistein | Human TRPV6 | Inhibitor | IC_50_ 40.7-113.2 | [48] | 8FOA | [48] |
| **6** | Central cavity site | Mg^2+^ | Mouse TRPM7 | Permeant ion | Working concentration: 300 000 | [85] | 6BWD | [85] |
| **6** | Central cavity site | Trpvicin | Human TRPV3 | Antagonist | IC_50_ 0.41 | [80] | 7XJ1  7XJ2 | [80] |
| **7** | Intracellular pore entry site | Ca^2+^ | Human PC2 | Agonist, channel blocker | Working concentration: 2 500 | [84] | 5MKE  5MKF | [84] |
| **7** | Intracellular pore entry site | CaM | Human TRPV5 | Inactivator, channel blocker | Working concentration: 2:1-1:1 TRPV5:CaM molar ratio | [39] | 5OEO | [39] |
| **7** | Intracellular pore entry site | CaM | Human TRPV6 | Inactivator, channel blocker | Working concentration: 20 | [30] | 6E2F | [30] |
| **7** | Intracellular pore entry site | CaM | Rabbit TRPV5 | Inactivator, channel blocker | Working concentration: 1:2-1:5 TRPV5:CaM moalr ratio | [29,31,32] | 6DMW  6O20  7T6R | [29,31,32] |
| **7** | Intracellular pore entry site | CaM | Rat TRPV1-ARD | Inactivator, channel blocker | Working concentration: 1.25:1–2.0:1 TRPV1:CaM | [91] | 3SUI | [91] |
| **7** | Intracellular pore entry site | CaM | Rat TRPV6 | Inactivator, channel blocker | Endogenous | [30] | 6E2G | [30] |
| **7** | Intracellular pore entry site | Mg^2+^ | Mouse TRPM7 | Permeant ion | Working concentration: 300 000 | [85] | 6BWD | [85] |
| **7** | Intracellular pore entry site | PCHPD (Br-cis-22a) | Rat TRPV6 | Inhibitor | IC_50_ 0.96 ± 0.03 54 | [67] | 7D2K | [67] |
| **7** | Intracellular pore entry site | PCHPD s (cis-22a, Br-cis-22a, 3OG, 30, 31) | Human TRPV6 | Inhibitors | IC_50_ 0.08 - 1.7 | [67,71-73] | 7K4B  7K4C  7K4D  7K4E  7K4F | [67] |
| **7** | Intracellular pore entry site | Genistein | Human TRPV6 | Inhibitor | IC_50_ 40.7-113.2 | [48] | 8FOA | [48] |
| **8** | Deep S4-S5 site | Piperlongumine (PL) | Rat TRPV2 | Antagonist | IC_50_ 4.6 ± 0.13 | [92] | 6WKN | [92] |
| **9** | Shallow S4-S5 site | 2-APB | Rat TRPV2 | Agonist | EC_50_ 322 ± 27 | [16] | 7N0M 8FFM | [16,90] |
| **9** | Shallow S4-S5 site | Econazole | Human TRPV6 | Inhibitor | IC_50_ 4.39 ± 0.31 | [47] | 7S8C | [47] |
| **9** | Shallow S4-S5 site | Econazole | Rabbit TRPV5 | Inhibitor | IC_50_ 1.3–2.0 | [69] | 8TF3  8TF4 | [69] |
| **10** | Deep portal site | Cannabidiol (CBD) | Rat TRPV2 | Agonist | EC_50_ 3.7 | [93] | 6U88 6U8A 7T37 8SLX | [16,93,94] |
| **10** | Deep portal site | Dyclonine | Mouse TRPV3 | Inhibitor | IC_50_ 29.8 ± 5.3 | [95] | 7UGG | [95] |
| **10** | Deep portal site | GDC-0334 | Human TRPA1 | Inhibitor | IC_50_ 0.0017 | [96] | 6WJ5 | [96] |
| **10** | Deep portal site | HC-070 | Human TRPC5 | Inhibitor | IC_50_ 0.2043 | [97] | 7D4Q | [97] |
| **10** | Deep portal site | ML-SA1 | Human TRPML1 | Agonist | EC_50_ 9.7-15.3 | [98] | 5WJ9 6E7Z | [14,99] |
| **10** | Deep portal site | ML-SA1 | Mouse TRPML1 | Agonist | Working concentration: 300 | [27] | 7SQ6 | [27] |
| **10** | Deep portal site | ML-SI3 | Human TRPML1 | Competitive antagonist | IC_50_ 3.9 | [98] | 7MGL | [98] |
| **10** | Deep portal site | Pico145 (HC-608) | Human TRPC5 | Inhibitor | IC_50_ 0.0013-0.0018 | [100,101] | 6YSN | [100] |
| **10** | Deep portal site | Temsirolimus | Mouse TRPML1 | Agonist | Working concentration: 500 | [27] | 7SQ9 | [27] |
| **10** | Deep portal site | THCV | Human TRPV6 | Inhibitor | IC_50_ 15.4 ± 2.3 | [49] | 8SP8 | [49] |
| **10** | Deep portal site | Cholesterol | Rat TRPV1 | - | Working concentration: 8% | [19] | 8U2Z | [19] |
| **11** | Shallow portal site | THCV | Human TRPV6 | Inhibitor | IC_50_ 15.4 ± 2.3 | [49] | 8SP8 | [49] |
| **11** | Shallow portal site | ZINC17988990 | Rabbit TRPV5 | Inhibitor | IC_50_ 0.106 ± 0.027 | [46] | 6PBE | [46] |
| **11** | Shallow portal site | ZINC9155420 | Rabbit TRPV5 | Inhibitor | IC_50_ 2.91 ± 0.56 | [46] | 6PBF | [46] |
| **12** | S1-S4 top site | 2-APB | Mouse TRPV3 | Agonist | EC_50_ 9-34 | [102-105] | 6DVY  6DVZ | [103] |
| **13** | S1-S4 base site | 2-APB | Human TRPV3 | Agonist | EC_50_ 28-93 | [9,104,106] | 8V6N | [9] |
| **13** | S1-S4 base site | 2-APB | Human TRPV6 | Inhibitor | IC_50_ 274 ± 27 | [82] | 6D7T | [82] |
| **13** | S1-S4 base site | 2-APB (2-APB-Br) | Rat TRPV6 | Inhibitor | IC_50_ 184 ± 8 | [82] | 6D7O  6D7Q  6D7V  6D7X | [82] |
| **13** | S1-S4 base site | 2-APB | Mouse TRPV3 | Agonist | EC_50_ 9-34 | [102-105] | 6DVY  6DVZ | [103] |
| **13** | S1-S4 base site | 4α-PDD | Human TRPV4 | Agonist | EC_50_ 0.45 ± 0.039 | [34] | 8FCA  8T1D | [33,34] |
| **13** | S1-S4 base site | Agonist C3 (Cryosim-3) | Mouse TRPM8 | Agonist | EC_50_ 10.3 ± 0.4 | [17] | 8E4M  8E4L | [17] |
| **13** | S1-S4 base site | Agonist-1 | Mouse TRPV4 | Agonist | EC_50_ 0.4 ± 0.1 | [45] | 8J1H | [45] |
| **13** | S1-S4 base site | Antagonist A1 | Human TRPV4 | Antagonist | IC_50_ 0.00253 ± 0.00052 | [35] | 8JU5 | [35] |
| **13** | S1-S4 base site | Antagonist A2 | Human TRPV4 | Antagonist | IC_50_ 0.00011 ± 0.00002 | [35] | 8JVI  8JVJ | [35] |
| **13** | S1-S4 base site | AM-1473 | Human TRPC6 | Antagonist | IC_50_ 0.00022 ± 0.00005 | [6] | 6UZA | [6] |
| **13** | S1-S4 base site | Clemizole | Human TRPC5 | Inhibitor | IC_50_ 3.03 | [97] | 7D4P | [97] |
| **13** | S1-S4 base site | GSK1016790A | Human TRPV4 | Agonist | EC_50_ 0.0021 | [107] | 8FC8  8FCB | [33] |
| **13** | S1-S4 base site | GSK1016790A | Mouse TRPV4 | Agonist | EC_50_ 1.6-2.8 | [45] | 8J1B  8J1F | [45] |
| **13** | S1-S4 base site | GSK2798745 | Human TRPV4 | Antagonist | IC_50_ 0.002-0.004 | [108] | 8FC7  8JU6 | [33] |
| **13** | S1-S4 base site | HC-067047 | Human TRPV4 | Inhibitor | IC_50_ 0.025 ± 0.0062 | [34] | 8T1F | [34] |
| **13** | S1-S4 base site | Icilin | Flycatcher TRPM8-A805G | Agonist | Working concentration: 200 | [8] | 6NR3 | [8] |
| **13** | S1-S4 base site | Icilin | Mouse TRPM8 | Agonist | Working concentration: 200 | [26] | 7WRD 7WRE 7WRF | [26] |
| **13** | S1-S4 base site | GFB-8438 | Zebrafish TRPC4 | Inhibitor | IC_50_ 1.8 ± 0 | [36] | 7B0S | [36] |
| **13** | S1-S4 base site | GFB-8749 | Zebrafish TRPC4 | Inhibitor | IC_50_ 0.16 ± 0.15 | [36] | 7B05 | [36] |
| **13** | S1-S4 base site | GFB-9289 | Zebrafish TRPC4 | Inhibitor | IC_50_ 25.2 ± 4.6 | [36] | 7B16 | [36] |
| **13** | S1-S4 base site | Osthole | Mouse TRPV3 | Competitive antagonist | IC_50_ 20-37 | [109,110] | 7RAS  7RAU | [110] |
| **13** | S1-S4 base site | Dic8-PI(3,5)P_2_ | Human TRPML1 | Agonist | Working concentration: 200 | [14] | 6E7P  6E7Z | [14] |
| **13** | S1-S4 base site | Dic8-PI(3,5)P_2_ | Mouse TRPML1 | Agonist | EC_50_ 0.048 | [111] | 7SQ7  7SQ9 | [27] |
| **13** | S1-S4 base site | DiC8-PI(4,5)P_2_ | Human TRPML1 | Competitive antagonist | Working concentration: 200 | [14] | 6E7Y | [14] |
| **13** | S1-S4 base site | Riluzole | Human TRPC5 | Activator | EC_50_ 9.2-20.7 in the presence of extracellular Ca^2+^ | [7,112] | 7WDB | [7] |
| **13** | S1-S4 base site | SAR7334 | Human TRPC6 | Inhibitor | IC_50_ 0.0095 | [113] | 7DXG | [5] |
| **13** | S1-S4 base site | ZINC17988990 | Rabbit TRPV5 | Inhibitor | IC_50_ 0.106 ± 0.027 | [46] | 6PBE | [46] |
| **13** | S1-S4 base site | WS-12 | Flycatcher TRPM8 | Agonist | Working concentration: 200 | [8] | 6NR2 | [8] |
| **14** | ARD-TMD linker site | 2-APB | Human TRPV3 | Agonist | EC_50_ 28-93 | [9,104,106] | 6OT5 8V6N | [9,114] |
| **14** | ARD-TMD linker site | 2-APB | Mouse TRPV3 | Agonist | EC_50_ 9-34 | [102-105] | 6DVY  6DVZ | [103] |
| **14** | ARD-TMD linker site | Osthole | Mouse TRPV3 | Competitive antagonist | IC_50_ 20-37 | [109,110] | 7RAS  7RAU | [110] |
| **15** | S2-S3 site | DiC8 PI(4,5)P_2_ | Rabbit TRPV5 | Endogenous activator/partial agonist | Working concentration: 200-400 | [29,32,69,115] | 6DMU  7T6M  7T6Q  8FFO  8TF4 | [29,32,69,115] |
| **15** | S2-S3 site | Oleoyl coenzyme A | Rabbit TRPV5 | Activator | Working concentration: 400 | [115] | 8FHI | [115] |
| **16** | ARD site | ATP | Human TRPV4 | Positive allosteric modulator | Working concentration: 5 000 | [116] | 4DX2 | [116] |
| **16** | ARD site | ATP | Rat TRPV1 | Positive allosteric modulator | Working concentration: 5 000 | [117] | 2NYJ  2PNN | [117] |
| **16** | ARD site | Destiobiotin | Rat TRPV6 | Unknown | Working concentration: 2 500 | [18] | 5IWT  5WO6  5WO7  5WO8  5WO9 | [18,83] |
| **17** | Lower central cavity site | Genistein | Human TRPV6 | Inhibitor | IC_50_ 40.7-113.2 | [48] | 8FOA | [48] |
| **18** | RhoA site | RhoA | Human TRPV4 | Inhibitor | Endogenous | [33-35] | 8FC7 8FC9 8FCB 8JVJ 8T1C | [33-35] |
| **TRPA new sites** | | | | | | | | |
| **19** | S4-S5 linker - TRP helix site (TRPA1 antagonist) | Antagonist compound 21 | Human TRPA1 | Antagonist | IC_50_ 0.0076 | [118] | 7JUP | [118] |
| **20** | S4-S5 linker site | Antagonist 3-60 | Human TRPA1 | Antagonist | IC_50_ 2.17 – 3.09 | [119] | 7OR0  7OR1 | To be published |
| **20** | S4-S5 linker site | CHS | Human TRPC3 | Structural lipid | Working concentration: 0.1% | [5] | 7DXB  7DXC  7DXD  7DXE | [5] |
| **20** | S4-S5 linker site | CHS | Human TRPC5 | Structural lipid | Working concentration: 0.01-0.1% | [7,37,97] | 7D4P  7D4Q  7E4T  7WDB  7X6C  7X6I  8GVW  8GVX | [7,37,97] |
| **20** | S4-S5 linker site | CHS | Human TRPC6 | Structural lipid | Working concentration: 0.1% | [5,6] | 6UZ8  6UZA  7DXF  7DXG | [5,6] |
| **20** | S4-S5 linker site | CHS | Mouse TRPC4 | Structural lipid | Working concentration: 0.01-0.1% | [120] | 5Z96  6JZO | [120] |
| **20** | S4-S5 linker site | CHS | Mouse TRPC5 | Structural lipid | Working concentration: 0.01-0.1% | [121] | 6AEI | [121] |
| **20** | S4-S5 linker site | CHS | Zebrafish TRPC4 | Structural lipid | Working concentration: 0.0026-0.1% | [122] | 6G1K | [122] |
| **20** | S4-S5 linker site | CHS | Tit TRPM8 | Stabilizer | Working concentration: 100-2 000 | [4] | 6O6A  6O6R  6O72  6O77 | [4] |
| **20** | S4-S5 linker site | DiC8-PI(4,5)P_2_ | Alga TRP-like ion channel | Agonist | Working concentration: 2.5 | [123] | 6PW4  6PW5 | [123] |
| **20** | S4-S5 linker site | DiC8-PI(4,5)P_2_ | Flycatcher TRPM8 | Allosteric modulator (positive) | Working concentration: 1 000 | [8,17] | 6NR2  6NR3  8E4Q | [8,17] |
| **20** | S4-S5 linker site | DiC8-PI(4,5)P_2_ | Mouse TRPM3 | Allosteric modulator (positive) | EC_50_ 18.01 | [124] | 8DDS  8DDT  8DDU  8DDV  8DDX  8ED8  8ED9 | [25] |
| **20** | S4-S5 linker site | DiC8-PI(4,5)P_2_ | Mouse TRPM8 | Allosteric modulator (positive) | Working concentration: 500-1 000 | [17,26] | 8E4L 8E4M 8E4N 8E4O | [17,26] |
| **21** | Coupling domain site | BITC | Human TRPA1 | Covalent agonist | Working concentration: 1 000 | [125] | 6PQP | [125] |
| **21** | Coupling domain site | Bodipy-iodoacetamide | Human TRPA1 | Covalent agonist | Working concentration: 100 | [54] | 6V9V | [54] |
| **21** | Coupling domain site | Iodoacetamide | Human TRPA1 | Covalent agonist | Working concentration: 100 | [54] | 6V9X | [54] |
| **21** | Coupling domain site | JT010 | Human TRPA1 | Covalent agonist | EC_50_ 0.0024-0.024 (0.308) | [125,126] | 6PQO | [125] |
| **22** | S2-S3 calcium site | Ca^2+^ | Flycatcher TRPM8 | Allosteric modulator (positive) | Working concentration: 1 000 | [8] | 6NR3 | [8] |
| **22** | S2-S3 calcium site | Ca^2+^ | Human TRPA1 | Allosteric modulator (positive and negative) | Working concentration: 2 000 | [54] | 6V9W 7OR1 | [54] To be published |
| **22** | S2-S3 calcium site | Ca^2+^ | Human TRPC3 | Activatory site | IC_50_ 0.8377-1.373 | [5] | 7DXB | [5] |
| **22** | S2-S3 calcium site | Ca^2+^ | Human TRPC5 | Activatory site | Working concentration: 100-5 000 | [7,37,97] | 7D4P  7D4Q  7E4T  7WDB  7X6C  7X6I  8GVW  8GVX | [7,37,97] |
| **22** | S2-S3 calcium site | Ca^2+^ | Human TRPC6 | Activatory site | Working concentration: 1 000 | [5] | 7DXF | [5] |
| **22** | S2-S3 calcium site | Ca^2+^ | Human TRPM2 | Agonist | EC_50_ 0.34 | [127] | 6MJ2  6PUS  6PUU | [128,129] |
| **22** | S2-S3 calcium site | Ca^2+^ | Human TRPM4 | Agonist | EC_50_ 4.4-524 | [130,131] | 9B8W  9B8X  9B8Y  9B8Z  9B90  9B91  9B92  9B94 | [12] |
| **22** | S2-S3 calcium site | Ca^2+^ | Mouse TRPC4 | - | - | - | 6JZO | To be published |
| **22** | S2-S3 calcium site | Ca^2+^ | Mouse TRPC5 | Unknown | Contaminant / endogenous | [121] | 6AEI | [121] |
| **22** | S2-S3 calcium site | Ca^2+^ | Mouse TRPM8 | Desensitizer, permeant ion | Working concentration: 500 | [26] | 7WRC  7WRD  7WRE  7WRF  8E4L  8E4M | [17,26] |
| **22** | S2-S3 calcium site | Ca^2+^ | Rat TRPM5 | Activator and desensitizer | Working concentration: 2 000 | [132] | 8SLI  8SLP  8SLW | [132] |
| **22** | S2-S3 calcium site | Ca^2+^ | Tit TRPM8 | Desensitizer | Working concentration: 2 000 | [4] | 6O6R  6O72  6O77 | [4] |
| **22** | S2-S3 calcium site | Ca^2+^ | Zebrafish TRPC4 | - | Working concentration: 10 000 | [36] | 7B05  7B0J  7B0S  7B16  7B1G | [36] |
| **22** | S2-S3 calcium site | Ca^2+^ | Zebrafish TRPM2 | Co-agonist | Working concentration: 500-2 000 | [43,133] | 6D73  6DRJ  6PKX | [43,133] |
| **22** | S2-S3 calcium site | Ca^2+^ | Zebrafish TRPM5 | Agonist | Working concentration: 6-5 000 | [78] | 7MBQ  7MBS  7MBU  7MBV | [78] |
| **TRPM new sites** | | | | | | | | |
| **23** | MHR1/2 site | ADPR | Human TRPM2 | Agonist | EC_50_ 77 | [134] | 6PUR  6PUS  6PUU | [129] |
| **23** | MHR1/2 site | 8-Br-cADPR | Human TRPM2 | Competitive antagonist | EC_50_ 251.1 ± 17.8 | [135] | 6PUU | [129] |
| **23** | MHR1/2 site | ADPR (ADPR-Br) | Zebrafish TRPM2 | Co-agonist | Working concentration: 50-1 000 | [43,133] | 6DRJ  6PKX | [43,133] |
| **24** | NUDT9-H site | ADPR | Human TRPM2 | Agonist | Kd 70.2 ± 10 | [135] | 6PUR  6PUS | [129] |
| **25** | Gβγ site (TRPM3) | Gβγ | Mouse TRPM3 | Inhibitory regulator | IC_50_ 0.24 ± 0.05 | [25] | 8DDW  8DDX | [25] |
| **26** | Rib helix junction site | Decavanadate | Human TRPM4 | Agonist | EC_50_ 1.9 | [130] | 5WP6 | [136] |
| **27** | MHR1/2-MHR3 interface site | ATP | Mouse TRPM4 | Inhibitor | IC_50_ 2.3 ± 0.5 | [137] | 6BCO  6BCQ | [137] |
| **27** | MHR1/2-MHR3 interface site | Decavanadate | Human TRPM4 | Agonist | EC_50_ 1.9 | [130] | 5WP6 | [136] |
| **28** | MHR calcium site | Ca^2+^ | Human TRPM4 | Agonist | EC_50_ 4.4-524 | [130,131] | 9B8W  9B8X  9B8Y  9B8Z | [12] |
| **28** | MHR calcium site | Ca^2+^ | Rat TRPM5 | Activator and desensitizer | Working concentration: 2 000 | [132] | 8SLI  8SLP  8SLW | [132] |
| **28** | MHR calcium site | Ca^2+^ | Zebrafish TRPM5 | Agonist | Working concentration: 6-5 000 | [78] | 7MBQ  7MBS | [78] |
| **29** | DVT warm site | Decavanadate | Human TRPM4 | Agonist | EC_50_ 1.9 | [130] | 9B8Y  9B8Z | [12] |
| **30** | MHR ATP site | ATP | Human TRPM4 | Inhibitor | IC_50_ 1.7±0.3 | [138] | 9B90  9B91 | [12] |
| **31** | NTB site | Naltriben | Mouse TRPM7 | Agonist | EC_50_ 65.8 ± 12.36 | [11] | 8SI5 | [11] |
| **TRPC new sites** | | | | | | | | |
| **32** | Top S4, S6 site | Cholesterol | Human PC2 | Structural lipid | Endogenous | [87] | 6T9N  6T9O | [87] |
| **32** | Top S4, S6 site | CHS | Human TRPC6 | Structural lipid | Working concentration: 0.1% | [5] | 7DXF  7DXG | [5] |
| **32** | Top S4, S6 site | AM-0883 | Human PC2 F604P | Agonist | EC_50_ 5.2 | [139] | 8HK7 | [139] |
| **33** | Top portal site | AM-0883 | Human TRPC6 (Δ2–72) | Agonist | EC_50_ 0.045 ± 0.01 for WT EC_50_ 0.0902 ± 0.013 for (Δ2–72) | [6] | 6UZ8 | [6] |
| **34** | Top S2-S3 site (TRPC CHS) | CHS | Human TRPC3 | Structural lipid | Working concentration: 0.1% | [5] | 7DXB  7DXC  7DXE | [5] |
| **34** | Top S2-S3 site (TRPC CHS) | CHS | Human TRPC6 | Structural lipid | Working concentration: 0.1% | [5] | 6UZ8  6UZA  7DXF  7DXG | [5,6] |
| **35** | Top S3-S4 site (TRPC6 CHS) | CHS | Human TRPC6 | Structural lipid | Working concentration: 0.1% | [5] | 7DXG | [5] |
| **36** | Top calcium site, CBS2 (TRPC) | Ca^2+^ | Human TRPC3 | Non-regulatory site | Working concentration: 1 000 | [5] | 7DXB | [5] |
| **36** | Top calcium site, CBS2 (TRPC) | Ca^2+^ | Human TRPC6 | Non-regulatory site | Working concentration: 1 000 | [5] | 7DXF  7DXG | [5] |
| **37** | Bottom calcium site, CBS1 (TRPC) | Ca^2+^ | Human TRPC3 | Inhibitory site | IC_50_ 0.8377-1.373 | [5] | 7DXB  7DXD | [5] |
| **37** | Bottom calcium site, CBS1 (TRPC) | Ca^2+^ | Human TRPC6 | Inhibitory site | Working concentration: 1 000 | [5] | 7DXF  7DXG | [5] |
| **38** | Calmodulin TRPC site | CaM | Zebrafish TRPC4 | Inhibitor | Working concentration: 10 | [36] | 7B1G | [36] |
| **39** | Zn site (TRPC) | Zn^2+^ | Human TRPC3 | - | - | - | 7DXB  7DXC  7DXD  7DXE | [5] |
| **39** | Zn site (TRPC) | Zn^2+^ | Human TRPC5 | - | - | - | 7D4P  7D4Q  7E4T  7WDB  7X6C  7X6I  8GVW  8GVX | [7,97] |
| **39** | Zn site (TRPC) | Zn^2+^ | Human TRPC6 | - | - | - | 7DXF  7DXG | [5] |
| **40** | Gα_i3_ site | G alpha i3 subunit, Q204L | Human TRPC5 | Allosteric modulator (positive) | EC_50_ 0.91 ± 0.01 | [37] | 7X6I  8GVX | [37] |

55. Vriens J, Appendino G, Nilius B: **Pharmacology of Vanilloid Transient Receptor Potential Cation Channels**. *Molecular Pharmacology* 2009, **75**:1262-1279.

56. Laursen WJ, Schneider ER, Merriman DK, Bagriantsev SN, Gracheva EO: **Low-cost functional plasticity of TRPV1 supports heat tolerance in squirrels and camels**. *Proceedings of the National Academy of Sciences* 2016, **113**:11342-11347.

57. Caterina MJ, Schumacher MA, Tominaga M, Rosen TA, Levine JD, Julius D: **The capsaicin receptor: a heat-activated ion channel in the pain pathway**. *Nature* 1997, **389**:816-824.

58. McIntyre P, McLatchie LM, Chambers A, Phillips E, Clarke M, Savidge J, Toms C, Peacock M, Shah K, Winter J, et al.: **Pharmacological differences between the human and rat vanilloid receptor 1 (VR1)**. *British Journal of Pharmacology* 2001, **132**:1084-1094.

59. Cao E, Liao M, Cheng Y, Julius D: **TRPV1 structures in distinct conformations reveal activation mechanisms**. *Nature* 2013, **504**:113-118.

60. Szallasi A, Blumberg PM, Annicelli LL, Krause JE, Cortright DN: **The Cloned Rat Vanilloid Receptor VR1 Mediates Both R-Type Binding and C-Type Calcium Response in Dorsal Root Ganglion Neurons**. *Molecular Pharmacology* 1999, **56**:581-587.

61. Zhang F, Hanson SM, Jara-Oseguera A, Krepkiy D, Bae C, Pearce LV, Blumberg PM, Newstead S, Swartz KJ: **Engineering vanilloid-sensitivity into the rat TRPV2 channel**. *Elife* 2016, **5**.

62. Zhang K, Julius D, Cheng Y: **Structural snapshots of TRPV1 reveal mechanism of polymodal functionality**. *Cell* 2021, **184**:5138-5150.e5112.

63. Zubcevic L, Le S, Yang H, Lee SY: **Conformational plasticity in the selectivity filter of the TRPV2 ion channel**. *Nat Struct Mol Biol* 2018, **25**:405-415.

64. Bevan S, Hothi S, Hughes G, James IF, Rang HP, Shah K, Walpole CSJ, Yeats JC: **Capsazepine: a competitive antagonist of the sensory neurone excitant capsaicin**. *British Journal of Pharmacology* 1992, **107**:544-552.

65. Cao E, Cordero-Morales Julio F, Liu B, Qin F, Julius D: **TRPV1 Channels Are Intrinsically Heat Sensitive and Negatively Regulated by Phosphoinositide Lipids**. *Neuron* 2013, **77**:667-679.

66. Nieto-Posadas A, Picazo-Juárez G, Llorente I, Jara-Oseguera A, Morales-Lázaro S, Escalante-Alcalde D, Islas LD, Rosenbaum T: **Lysophosphatidic acid directly activates TRPV1 through a C-terminal binding site**. *Nature Chemical Biology* 2012, **8**:78-85.

67. Bhardwaj R, Lindinger S, Neuberger A, Nadezhdin KD, Singh AK, Cunha MR, Derler I, Gyimesi G, Reymond J-L, Hediger MA, et al.: **Inactivation-mimicking block of the epithelial calcium channel TRPV6**. *Science Advances* 2020, **6**:eabe1508.

68. Hughes TET, Lodowski DT, Huynh KW, Yazici A, Del Rosario J, Kapoor A, Basak S, Samanta A, Han X, Chakrapani S, et al.: **Structural basis of TRPV5 channel inhibition by econazole revealed by cryo-EM**. *Nature Structural & Molecular Biology* 2018, **25**:53-60.

69. De Jesús-Pérez JJ, Gabrielle M, Raheem S, Fluck EC, Rohacs T, Moiseenkova-Bell VY: **Structural mechanism of TRPV5 inhibition by econazole**. *Structure* 2024, **32**:148-156.e145.

70. Nilius B, Prenen J, Vennekens R, Hoenderop JGJ, Bindels RJM, Droogmans G: **Pharmacological modulation of monovalent cation currents through the epithelial Ca ^2+^ channel ECaC1**. *British Journal of Pharmacology* 2001, **134**:453-462.

71. Simonin C, Awale M, Brand M, van Deursen R, Schwartz J, Fine M, Kovacs G, Häfliger P, Gyimesi G, Sithampari A, et al.: **Optimization of TRPV6 Calcium Channel Inhibitors Using a 3D Ligand‐Based Virtual Screening Method**. *Angewandte Chemie International Edition* 2015, **54**:14748-14752.

72. Cunha MR, Bhardwaj R, Carrel AL, Lindinger S, Romanin C, Parise-Filho R, Hediger MA, Reymond J-L: **Natural product inspired optimization of a selective TRPV6 calcium channel inhibitor**. *RSC Medicinal Chemistry* 2020, **11**:1032-1040.

73. Cunha MR, Bhardwaj R, Lindinger S, Butorac C, Romanin C, Hediger MA, Reymond J-L: **Photoswitchable Inhibitor of the Calcium Channel TRPV6**. *ACS Medicinal Chemistry Letters* 2019, **10**:1341-1345.

74. Tang Q, Guo W, Zheng L, Wu J-X, Liu M, Zhou X, Zhang X, Chen L: **Structure of the receptor-activated human TRPC6 and TRPC3 ion channels**. *Cell Research* 2018, **28**:746-755.

75. Janssens A, Gees M, Toth BI, Ghosh D, Mulier M, Vennekens R, Vriens J, Talavera K, Voets T: **Definition of two agonist types at the mammalian cold-activated channel TRPM8**. *eLife* 2016, **5**:e17240.

76. Nadezhdin KD, Correia L, Shalygin A, Aktolun M, Neuberger A, Gudermann T, Kurnikova MG, Chubanov V, Sobolevsky AI: **Structural basis of selective TRPM7 inhibition by the anticancer agent CCT128930**. *Cell Reports* 2024, **43**:114108.

77. Liu C, Reese R, Vu S, Rougé L, Shields SD, Kakiuchi-Kiyota S, Chen H, Johnson K, Shi YP, Chernov-Rogan T, et al.: **A Non-covalent Ligand Reveals Biased Agonism of the TRPA1 Ion Channel**. *Neuron* 2021, **109**:273-284.e274.

78. Ruan Z, Haley E, Orozco IJ, Sabat M, Myers R, Roth R, Du J, Lü W: **Structures of the TRPM5 channel elucidate mechanisms of activation and inhibition**. *Nature Structural & Molecular Biology* 2021, **28**:604-613.

79. Medley Q, YANG J, Demirs JT, Papillon J, Gao Y, Xu Y, Chastain J, Haque S, Palmer MM, Wrynn A: **In-vitro and In-vivo pharmacology of SAF312 as a TRPV1 inhibitor for ocular surface pain**. *Investigative Ophthalmology & Visual Science* 2021, **62**:722-722.

80. Fan J, Hu L, Yue Z, Liao D, Guo F, Ke H, Jiang D, Yang Y, Lei X: **Structural basis of TRPV3 inhibition by an antagonist**. *Nature Chemical Biology* 2023, **19**:81-90.

81. Bohlen CJ, Priel A, Zhou S, King D, Siemens J, Julius D: **A Bivalent Tarantula Toxin Activates the Capsaicin Receptor, TRPV1, by Targeting the Outer Pore Domain**. *Cell* 2010, **141**:834-845.

82. Singh AK, Saotome K, McGoldrick LL, Sobolevsky AI: **Structural bases of TRP channel TRPV6 allosteric modulation by 2-APB**. *Nature Communications* 2018, **9**:2465.

83. Singh AK, Saotome K, Sobolevsky AI: **Swapping of transmembrane domains in the epithelial calcium channel TRPV6**. *Scientific Reports* 2017, **7**:10669.

84. Wilkes M, Madej MG, Kreuter L, Rhinow D, Heinz V, De Sanctis S, Ruppel S, Richter RM, Joos F, Grieben M, et al.: **Molecular insights into lipid-assisted Ca2+ regulation of the TRP channel Polycystin-2**. *Nature Structural & Molecular Biology* 2017, **24**:123-130.

85. Duan J, Li Z, Li J, Hulse RE, Santa-Cruz A, Valinsky WC, Abiria SA, Krapivinsky G, Zhang J, Clapham DE: **Structure of the mammalian TRPM7, a magnesium channel required during embryonic development**. *Proceedings of the National Academy of Sciences* 2018, **115**.

86. Deng Z, Paknejad N, Maksaev G, Sala-Rabanal M, Nichols CG, Hite RK, Yuan P: **Cryo-EM and X-ray structures of TRPV4 reveal insight into ion permeation and gating mechanisms**. *Nature Structural & Molecular Biology* 2018, **25**:252-260.

87. Wang Q, Corey RA, Hedger G, Aryal P, Grieben M, Nasrallah C, Baronina A, Pike ACW, Shi J, Carpenter EP, et al.: **Lipid Interactions of a Ciliary Membrane TRP Channel: Simulation and Structural Studies of Polycystin-2**. *Structure* 2020, **28**:169-184.e165.

88. Botte M, Ulrich AKC, Adaixo R, Gnutt D, Brockmann A, Bucher D, Chami M, Bocquet N, Ebbinghaus-Kintscher U, Puetter V, et al.: **Cryo-EM structural studies of the agonist complexed human TRPV4 ion-channel reveals novel structural rearrangements resulting in an open-conformation**. 2020.

89. Hoenderop JG, Vennekens R, Muller D, Prenen J, Droogmans G, Bindels RJ, Nilius B: **Function and expression of the epithelial Ca(2+) channel family: comparison of mammalian ECaC1 and 2**. *J Physiol* 2001, **537**:747-761.

90. Pumroy RA, De Jesús-Pérez JJ, Protopopova AD, Rocereta JA, Fluck EC, Fricke T, Lee B-H, Rohacs T, Leffler A, Moiseenkova-Bell V: **Molecular details of ruthenium red pore block in TRPV channels**. *EMBO Reports* 2024, **25**:506-523.

91. Lau S-Y, Procko E, Gaudet R: **Distinct properties of Ca2+–calmodulin binding to N- and C-terminal regulatory regions of the TRPV1 channel**. *Journal of General Physiology* 2012, **140**:541-555.

92. Conde J, Pumroy RA, Baker C, Rodrigues T, Guerreiro A, Sousa BB, Marques MC, De Almeida BP, Lee S, Leites EP, et al.: **Allosteric Antagonist Modulation of TRPV2 by Piperlongumine Impairs Glioblastoma Progression**. *ACS Central Science* 2021, **7**:868-881.

93. Pumroy RA, Samanta A, Liu Y, Hughes TE, Zhao S, Yudin Y, Rohacs T, Han S, Moiseenkova-Bell VY: **Molecular mechanism of TRPV2 channel modulation by cannabidiol**. *eLife* 2019, **8**:e48792.

94. Gochman A, Tan X-F, Bae C, Chen H, Swartz KJ, Jara-Oseguera A: **Cannabidiol sensitizes TRPV2 channels to activation by 2-APB**. *eLife* 2023, **12**:e86166.

95. Neuberger A, Nadezhdin KD, Sobolevsky AI: **Structural mechanism of TRPV3 channel inhibition by the anesthetic dyclonine**. *Nature Communications* 2022, **13**:2795.

96. Balestrini A, Joseph V, Dourado M, Reese RM, Shields SD, Rougé L, Bravo DD, Chernov-Rogan T, Austin CD, Chen H, et al.: **A TRPA1 inhibitor suppresses neurogenic inflammation and airway contraction for asthma treatment**. *Journal of Experimental Medicine* 2021, **218**:e20201637.

97. Song K, Wei M, Guo W, Quan L, Kang Y, Wu J-X, Chen L: **Structural basis for human TRPC5 channel inhibition by two distinct inhibitors**. *eLife* 2021, **10**:e63429.

98. Schmiege P, Fine M, Li X: **Atomic insights into ML-SI3 mediated human TRPML1 inhibition**. *Structure* 2021, **29**:1295-1302.e1293.

99. Schmiege P, Fine M, Blobel G, Li X: **Human TRPML1 channel structures in open and closed conformations**. *Nature* 2017, **550**:366-370.

100. Wright DJ, Simmons KJ, Johnson RM, Beech DJ, Muench SP, Bon RS: **Human TRPC5 structures reveal interaction of a xanthine-based TRPC1/4/5 inhibitor with a conserved lipid binding site**. *Communications Biology* 2020, **3**:704.

101. Rubaiy HN, Ludlow MJ, Henrot M, Gaunt HJ, Miteva K, Cheung SY, Tanahashi Y, Hamzah N, Musialowski KE, Blythe NM, et al.: **Picomolar, selective, and subtype-specific small-molecule inhibition of TRPC1/4/5 channels**. *Journal of Biological Chemistry* 2017, **292**:8158-8173.

102. Hu H-Z, Gu Q, Wang C, Colton CK, Tang J, Kinoshita-Kawada M, Lee L-Y, Wood JD, Zhu MX: **2-Aminoethoxydiphenyl Borate Is a Common Activator of TRPV1, TRPV2, and TRPV3**. *Journal of Biological Chemistry* 2004, **279**:35741-35748.

103. Singh AK, McGoldrick LL, Sobolevsky AI: **Structure and gating mechanism of the transient receptor potential channel TRPV3**. *Nature Structural & Molecular Biology* 2018, **25**:805-813.

104. Chung M-K, Lee H, Mizuno A, Suzuki M, Caterina MJ: **2-Aminoethoxydiphenyl Borate Activates and Sensitizes the Heat-Gated Ion Channel TRPV3**. *The Journal of Neuroscience* 2004, **24**:5177-5182.

105. Hu H, Grandl J, Bandell M, Petrus M, Patapoutian A: **Two amino acid residues determine 2-APB sensitivity of the ion channels TRPV3 and TRPV4**. *Proceedings of the National Academy of Sciences* 2009, **106**:1626-1631.

106. Deering‐Rice CE, Mitchell VK, Romero EG, Abdel Aziz MH, Ryskamp DA, Križaj D, Venkat RG, Reilly CA: **Drofenine: a 2‐APB analog with improved selectivity for human TRPV3**. *Pharmacology Research & Perspectives* 2014, **2**:e00062.

107. Thorneloe KS, Sulpizio AC, Lin Z, Figueroa DJ, Clouse AK, McCafferty GP, Chendrimada TP, Lashinger ESR, Gordon E, Evans L, et al.: **N-((1S)-1-{[4-((2S)-2-{[(2,4-Dichlorophenyl)sulfonyl]amino}-3-hydroxypropanoyl)-1-piperazinyl]carbonyl}-3-methylbutyl)-1-benzothiophene-2-carboxamide (GSK1016790A), a Novel and Potent Transient Receptor Potential Vanilloid 4 Channel Agonist Induces Urinary Bladder Contraction and Hyperactivity: Part I**. *Journal of Pharmacology and Experimental Therapeutics* 2008, **326**:432-442.

108. Goyal N, Skrdla P, Schroyer R, Kumar S, Fernando D, Oughton A, Norton N, Sprecher DL, Cheriyan J: **Clinical Pharmacokinetics, Safety, and Tolerability of a Novel, First-in-Class TRPV4 Ion Channel Inhibitor, GSK2798745, in Healthy and Heart Failure Subjects**. *American Journal of Cardiovascular Drugs* 2019, **19**:335-342.

109. Sun X-Y, Sun L-L, Qi H, Gao Q, Wang G-X, Wei N-N, Wang K: **Antipruritic Effect of Natural Coumarin Osthole through Selective Inhibition of Thermosensitive TRPV3 Channel in the Skin**. *Molecular Pharmacology* 2018, **94**:1164-1173.

110. Neuberger A, Nadezhdin KD, Zakharian E, Sobolevsky AI: **Structural mechanism of TRPV3 channel inhibition by the plant‐derived coumarin osthole**. *EMBO reports* 2021, **22**:e53233.

111. Dong X-p, Shen D, Wang X, Dawson T, Li X, Zhang Q, Cheng X, Zhang Y, Weisman LS, Delling M, et al.: **PI(3,5)P2 controls membrane trafficking by direct activation of mucolipin Ca2+ release channels in the endolysosome**. *Nature Communications* 2010, **1**:38.

112. Richter JM, Schaefer M, Hill K: **Riluzole activates TRPC5 channels independently of PLC activity**. *British Journal of Pharmacology* 2014, **171**:158-170.

113. Maier T, Follmann M, Hessler G, Kleemann H-W, Hachtel S, Fuchs B, Weissmann N, Linz W, Schmidt T, Löhn M, et al.: **Discovery and pharmacological characterization of a novel potent inhibitor of diacylglycerol-sensitive TRPC cation channels**. *British Journal of Pharmacology* 2015, **172**:3650-3660.

114. Zubcevic L, Borschel WF, Hsu AL, Borgnia MJ, Lee S-Y: **Regulatory switch at the cytoplasmic interface controls TRPV channel gating**. *eLife* 2019, **8**:e47746.

115. Lee B-H, De Jesús Pérez JJ, Moiseenkova-Bell V, Rohacs T: **Structural basis of the activation of TRPV5 channels by long-chain acyl-Coenzyme-A**. *Nature Communications* 2023, **14**:5883.

116. Inada H, Procko E, Sotomayor M, Gaudet R: **Structural and Biochemical Consequences of Disease-Causing Mutations in the Ankyrin Repeat Domain of the Human TRPV4 Channel**. *Biochemistry* 2012, **51**:6195-6206.

117. Lishko PV, Procko E, Jin X, Phelps CB, Gaudet R: **The Ankyrin Repeats of TRPV1 Bind Multiple Ligands and Modulate Channel Sensitivity**. *Neuron* 2007, **54**:905-918.

118. Terrett JA, Chen H, Shore DG, Villemure E, Larouche-Gauthier R, Déry M, Beaumier F, Constantineau-Forget L, Grand-Maître C, Lépissier L, et al.: **Tetrahydrofuran-Based Transient Receptor Potential Ankyrin 1 (TRPA1) Antagonists: Ligand-Based Discovery, Activity in a Rodent Asthma Model, and Mechanism-of-Action via Cryogenic Electron Microscopy**. *Journal of Medicinal Chemistry* 2021, **64**:3843-3869.

119. Saward BG: **Determining the role of the human transient receptor potential subfamily A member 1 (hTRPA1) ion channel in pain sensation**. Edited by: University of Oxford; 2021.

120. Duan J, Li J, Zeng B, Chen G-L, Peng X, Zhang Y, Wang J, Clapham DE, Li Z, Zhang J: **Structure of the mouse TRPC4 ion channel**. *Nature Communications* 2018, **9**:3102.

121. Duan J, Li J, Chen G-L, Ge Y, Liu J, Xie K, Peng X, Zhou W, Zhong J, Zhang Y, et al.: **Cryo-EM structure of TRPC5 at 2.8-Å resolution reveals unique and conserved structural elements essential for channel function**. *Science Advances* 2019, **5**:eaaw7935.

122. Vinayagam D, Mager T, Apelbaum A, Bothe A, Merino F, Hofnagel O, Gatsogiannis C, Raunser S: **Electron cryo-microscopy structure of the canonical TRPC4 ion channel**. *eLife* 2018, **7**:e36615.

123. McGoldrick LL, Singh AK, Demirkhanyan L, Lin T-Y, Casner RG, Zakharian E, Sobolevsky AI: **Structure of the thermo-sensitive TRP channel TRP1 from the alga Chlamydomonas reinhardtii**. *Nature Communications* 2019, **10**:4180.

124. Badheka D, Borbiro I, Rohacs T: **Transient receptor potential melastatin 3 is a phosphoinositide-dependent ion channel**. *Journal of General Physiology* 2015, **146**:65-77.

125. Suo Y, Wang Z, Zubcevic L, Hsu AL, He Q, Borgnia MJ, Ji R-R, Lee S-Y: **Structural Insights into Electrophile Irritant Sensing by the Human TRPA1 Channel**. *Neuron* 2020, **105**:882-894.e885.

126. Heber S, Gold-Binder M, Ciotu CI, Witek M, Ninidze N, Kress H-G, Fischer MJM: **A Human TRPA1-Specific Pain Model**. *The Journal of Neuroscience* 2019, **39**:3845-3855.

127. McHugh D, Flemming R, Xu S-Z, Perraud A-L, Beech DJ: **Critical Intracellular Ca2+ Dependence of Transient Receptor Potential Melastatin 2 (TRPM2) Cation Channel Activation**. *Journal of Biological Chemistry* 2003, **278**:11002-11006.

128. Wang L, Fu T-M, Zhou Y, Xia S, Greka A, Wu H: **Structures and gating mechanism of human TRPM2**. *Science* 2018, **362**:eaav4809.

129. Huang Y, Roth B, Lü W, Du J: **Ligand recognition and gating mechanism through three ligand-binding sites of human TRPM2 channel**. *eLife* 2019, **8**:e50175.

130. Nilius B, Prenen J, Janssens A, Voets T, Droogmans G: **Decavanadate modulates gating of TRPM4 cation channels**. *The Journal of Physiology* 2004, **560**:753-765.

131. Zhang Z, Okawa H, Wang Y, Liman ER: **Phosphatidylinositol 4,5-Bisphosphate Rescues TRPM4 Channels from Desensitization**. *Journal of Biological Chemistry* 2005, **280**:39185-39192.

132. Karuppan S, Schrag LG, Pastrano CM, Jara-Oseguera A, Zubcevic L: **Structural dynamics at cytosolic interprotomer interfaces control gating of a mammalian TRPM5 channel**. *Proceedings of the National Academy of Sciences* 2024, **121**:e2403333121.

133. Huang Y, Winkler PA, Sun W, Lü W, Du J: **Architecture of the TRPM2 channel and its activation mechanism by ADP-ribose and calcium**. *Nature* 2018, **562**:145-149.

134. Belrose JC, Xie Y-F, Gierszewski LJ, MacDonald JF, Jackson MF: **Loss of glutathione homeostasis associated with neuronal senescence facilitates TRPM2 channel activation in cultured hippocampal pyramidal neurons**. *Molecular Brain* 2012, **5**:11.

135. Yu P, Liu Z, Yu X, Ye P, Liu H, Xue X, Yang L, Li Z, Wu Y, Fang C, et al.: **Direct Gating of the TRPM2 Channel by cADPR via Specific Interactions with the ADPR Binding Pocket**. *Cell Reports* 2019, **27**:3684-3695.e3684.

136. Winkler PA, Huang Y, Sun W, Du J, Lu W: **Electron cryo-microscopy structure of a human TRPM4 channel**. *Nature* 2017, **552**:200-204.

137. Guo J, She J, Zeng W, Chen Q, Bai X-c, Jiang Y: **Structures of the calcium-activated, non-selective cation channel TRPM4**. *Nature* 2017, **552**:205-209.

138. Nilius B, Prenen J, Voets T, Droogmans G: **Intracellular nucleotides and polyamines inhibit the Ca2+-activated cation channel TRPM4b**. *Pflügers Archiv* 2004, **448**:70-75.

139. Wang Z, Chen M, Su Q, Morais TDC, Wang Y, Nazginov E, Pillai AR, Qian F, Shi Y, Yu Y: **Molecular and structural basis of the dual regulation of the polycystin-2 ion channel by small-molecule ligands**. *Proceedings of the National Academy of Sciences* 2024, **121**:e2316230121.
